# Comment on “The Effects of Various Essential Oils on Epilepsy and Acute Seizure: A Systematic Review”

**DOI:** 10.1155/2019/6829428

**Published:** 2019-09-30

**Authors:** Thomas Mathew, Saji K. John, Vikram Kamath, Asha Shaji

**Affiliations:** ^1^Department of Neurology, St. John's Medical College Hospital, Sarjapura Road, Bengaluru 560034, Karnataka, India; ^2^Department of Neurology, Apollo Hospitals, Bannerghatta Road, Bengaluru 560076, Karnataka, India

We have read with great interest the article by Bahr et al. entitled *The Effects of Various Essential Oils on Epilepsy and Acute Seizure: A Systematic Review*, published in May 2019 edition [1]. The article is very informative and is pertinent in the current context, as a lot of essential oils are used and misused by the public. The effects/outcome of various essential oils on epilepsy and acute seizure are well described in the article. As the authors have reviewed only the publications up to 2017, they have missed one important publication on seizures induced by the inhalation of the essential oil of eucalyptus. We, from Bangalore, South India, had described 10 cases of eucalyptus oil inhalation-induced seizures in 2018 [2]. We have also presented a paper on essential oil-related seizures due to balms and various preparations containing the mixture of essential oils of eucalyptus and camphor in a recently concluded American Epilepsy Society meeting in New Orleans [3]. Indeed, we observed that many of the cases of the so-called “idiopathic seizures” are induced and provoked by essential oils of eucalyptus and camphor as they are the most common commercially available essential oils. We have also observed that inhalation, ingestion, and even topical application can trigger seizure. In our case series, indeed, topical application was the commonest mode of exposure. Physicians are not aware of the proconvulsant properties of these essential oils and rarely enquire about the exposure to these in their history taking [4]. As all these balms, oils, and other products containing eucalyptus, camphor, and other epileptogenic essential oils are freely sold in the market and are used by many, thinking they are safe, it is high time that public, especially those with seizure and epilepsy, should be counselled to avoid these essential oils with proconvulsant properties [5]. Articles like this should sensitize commercial companies and regulatory authorities to put labels on products with proconvulsant essential oils stating “*potentially proconvulsant and to be avoided by people with epilepsy*.” This may prevent many cases of essential oil-related seizures, especially those secondary to usage of camphor and eucalyptus.
